# Measurement of hemoglobin in the operating room: what Methods can we trust?

**DOI:** 10.1186/cc11040

**Published:** 2012-03-20

**Authors:** B Giraud, D Frasca, O Mimoz

**Affiliations:** 1 CHU La Milétrie Poitiers, France

## Introduction

A noninvasive and continuous monitoring of total hemoglobin (Hb) by spectrophotometry was recently marketed (SpHb; Masimo, Irvine, CA, USA). The main objective of this study was to determine the absolute and trend accuracy of SpHb compared to Hb assessment at the laboratory (HbLab) used as the reference method.

## Methods

After obtaining ethics committee approval and informed consent, 51 adult patients (29 men, 22 women, age 18 to 90 years) undergoing major surgery with expected large blood loss were enrolled in the study. Patients wore Rainbow adult resposable sensors (R2-25, Revision E) connected to a Radical-7 Pulse CO-Oximeter, software version 7.6.0.1. HbLab values were obtained by analyzing arterial blood samples at the laboratory using a Sysmex XT-2100i automated hematology analyzer (Roche Diagnostics, Paris, France). The same samples were also analyzed with a satellite laboratory CO-Oximeter (Siemens RapidPoint 405 CO-Oximeter; Siemens, Munich, Germany), HbSat, and a point-of-care hemoglobinometer (HemoCue, Hb201; Ångelholm, Sweden), HcueArt. At the same time, a fourth drop of blood after skin puncture on the ear or finger was taken for capillary blood sampling tested also with the HemoCue: HcueCap. Invasive Hb values were compared to Sphb obtained at the time of the blood draw. An initial set was collected before surgery. Then blood samples were taken on approximately an hourly basis or more often if clinically indicated. Bland-Altman method plots were used to compare absolute accuracy of test devices to laboratory values. The ability of the test devices to follow the trend of the changes in Hb values reported by the reference device was assessed by plotting the difference between subsequent measurements reported by each device to the difference in subsequent measurements reported by the reference device, and a coefficient of determination (*R*^2^) was calculated.

## Results

The study included 210 measurements. HbLab ranged between 6.8 and 16.3 g/dl. Compared to the reference method, the average bias was 0.96 ± 2.78 g/dl for SpHb, 0.16 ± 0.45 g/dl for HcueArt, 0.5 ± 1.71 g/dl for HcueCap and 0.81 ± 1.04 g/dl for HbSat. *R*^2 ^values were 0.39 for SpHb, 0.93 for HcueArt, 0.53 for HcueCap and 0.47 for HbSat.

## Conclusion

This study shows that HcueArt seem the most reliable method of Hb assessment. The SpHb has a lower accuracy, but its ability to monitor Hb continuously and noninvasively remains attractive and development of this method should be encouraged.

